# Polygonogram and isobolographic analysis of interactions between various novel antiepileptic drugs in the 6-Hz corneal stimulation-induced seizure model in mice

**DOI:** 10.1371/journal.pone.0234070

**Published:** 2020-06-01

**Authors:** Jarogniew J. Luszczki, Anna Panasiuk, Mirosław Zagaja, Sławomir Karwan, Hubert Bojar, Zbigniew Plewa, Magdalena Florek-Łuszczki

**Affiliations:** 1 Department of Pathophysiology, Medical University of Lublin, Lublin, Poland; 2 Isobolographic Analysis Laboratory, Institute of Rural Health, Lublin, Poland; 3 Department of Anesthesiology and Intensive Care, Medical University of Lublin, Lublin, Poland; 4 Regional Specialized Children’s Hospital, Olsztyn, Poland; 5 Department of Toxicology and Food Safety, Institute of Rural Health, Lublin, Poland; 6 Department of General, Oncological and Minimally Invasive Surgery, 1st Military Clinical Hospital, Lublin, Poland; 7 Department of Medical Anthropology, Institute of Rural Health, Lublin, Poland; University of Modena and Reggio Emilia, ITALY

## Abstract

Pharmacotherapy with two antiepileptic drugs in combination is usually prescribed to epilepsy patients with refractory seizures. The choice of antiepileptic drugs in combination should be based on synergistic cooperation of the drugs with respect to suppression of seizures. The selection of synergistic interactions between antiepileptic drugs is challenging issue for physicians, especially, if 25 antiepileptic drugs are currently available and approved to treat epilepsy patients. The aim of this study was to determine all possible interactions among 5 second-generation antiepileptic drugs (gabapentin (GBP), lacosamide (LCM), levetiracetam (LEV), pregabalin (PGB) and retigabine (RTG)) in the 6-Hz corneal stimulation-induced seizure model in adult male albino Swiss mice. The anticonvulsant effects of 10 various two-drug combinations of antiepileptic drugs were evaluated with type I isobolographic analysis associated with graphical presentation of polygonogram to visualize the types of interactions. Isobolographic analysis revealed that 7 two-drug combinations of LEV+RTG, LEV+LCM, GBP+RTG, PGB+LEV, GBP+LEV, PGB+RTG, PGB+LCM were synergistic in the 6-Hz corneal stimulation-induced seizure model in mice. The additive interaction was observed for the combinations of GBP+LCM, GBP+PGB, and RTG+LCM in this seizure model in mice. The most beneficial combination, offering the highest level of synergistic suppression of seizures in mice was that of LEV+RTG, whereas the most additive combination that protected the animals from seizures was that reporting additivity for RTG+LCM. The strength of interaction for two-drug combinations can be arranged from the synergistic to the additive, as follows: LEV+RTG > LEV+LCM > GBP+RTG > PGB+LEV > GBP+LEV > PGB+RTG > PGB+LCM > GBP+LCM > GBP+PGB > RTG+LCM.

## Introduction

Patients with drug resistant epilepsy always need effective antiepileptic drugs that should suppress or reduce their epileptic attacks [1, 2]. Although clinicians can dispose up to 25 antiepileptic drugs, there is still a need to discover novel efficacious antiepileptic drugs [3]. In case of drug resistant epilepsy, which has recently been defined as “failure of adequate trials of two tolerated, appropriately chosen and used antiepileptic drug schedules (whether as monotherapies or in combination) to achieve sustained seizure freedom” [4], the patients can be treated with novel (second- and third-generation) antiepileptic drugs [5]. At present, no guidelines exist that could preferentially favor some combination therapies over monotherapy with antiepileptic drugs in epilepsy patients [2, 6].

Evidence-based medicine related with favorable antiepileptic drug combinations in epileptic patients is scarce or limited to only few antiepileptic drug combinations, whose clinical efficacy have been confirmed several years ago [7–9]. Undoubtedly, no novel antiepileptic drug combinations have recently been confirmed clinically as effective in terms of reduction of seizure activity in epileptic patients. Of note, each combination of antiepileptic drugs is closely associated with drug-drug interaction, whose nature may be additive, antagonistic, synergistic or neutral [10, 11]. Clinicians, when prescribing antiepileptic drugs in combination for their patients must consider several presumptions based primarily on various molecular mechanisms of action of the combined antiepileptic drugs, prognosis of the disease, tolerance of the antiepileptic drugs by the patients and adverse effects produced by the antiepileptic drugs [12, 13]. At present, there are no special recommendations about prescriptions of the most efficient antiepileptic drug combinations for patients with drug resistant epilepsy [2].

Researchers and clinicians are still searching for the best antiepileptic drug combinations, which could efficiently suppress seizures in patients with refractory epilepsy [13]. Presently, preclinical studies on animals provide us with valuable information about favorable antiepileptic drug combinations, which in animals offer synergistic interaction in terms of seizure suppression [14–18]. At present, in experimental epileptology only the isobolographic analysis of interaction provides the proper classification of types of interaction between antiepileptic drugs [14, 15]. Based on isobolographic analysis the most favorable antiepileptic drug combinations have been selected, but these combinations usually contain classical antiepileptic drugs [14]. It is widely accepted that the second- and third-generation antiepileptic drugs possess more favorable pharmacokinetic profiles than classical antiepileptic drugs do [19, 20]. The clinical usage of second- and third-generation antiepileptic drugs is usually associated with low risk of adverse effects evoked by these antiepileptic drugs in epileptic patients [21]. Unfortunately, favorable combinations of antiepileptic drugs exclusively comprised the second- and third-generation antiepileptic drugs are scarce [22–28]. To ensure the patients with refractory epilepsy with the best treatment options, experiments on animals should verify, which of the tested antiepileptic drug combinations are beneficial and exert synergistic interactions with respect to their anticonvulsant effects that would be transferred to clinical conditions.

The aim of this study was to determine the types of interactions between several antiepileptic drugs belonging to the second-generations of antiepileptic drugs, namely, gabapentin (GBP), lacosamide (LCM), levetiracetam (LEV), pregabalin (PGB), and retigabine (RTG). Evaluation of the anticonvulsant properties of the investigated antiepileptic drugs was performed in mice subjected to the 6-Hz corneal stimulation-induced seizure model, which is thought to be an experimental model of psychomotor (limbic) seizures in humans [29, 30]. More specifically, various stereotypical and behavioral manifestations of seizure activity in animals, subjected to the electrical stimulation with current at frequency of 6 Hz, are similar to those observed in patients with partial (limbic) seizures [29, 30]. The selection of the antiepileptic drugs was based on their various different molecular mechanisms of action and favorable pharmacokinetic properties. Additionally, the selection of second-generation antiepileptic drugs was based on their low inclination to produce adverse (toxic) effects in both, experimental animals and epileptic patients receiving these antiepileptics. The high safety profiles of the selected antiepileptic drugs were also the criterion for selecting antiepileptic drugs to combination. To unequivocally assess the types of two-drug interactions between antiepileptic drugs, we tested all possible interactions that can be observed for these 5 antiepileptic drugs (GBP, LCM, LEV, PGB and RTG).

## Material and methods

### Animals

Adult male CD-1 (albino Swiss) outbred mice (8 week-old, weighing 20–25 g), used in this study were bred at the place, where the experiments were carried out i.e., at the Experimental Medicine Center of Medical University of Lublin, Poland. The animal CD-1 stock has been originally purchased several years ago from AnimaLab, Poznan, Poland (official distributor of Charles Rivers animals). The animals were kept in large shoe-box cages (20 mice per cage) with free access to food (Altromin^®^, Maintenance diet for rats and mice—Totally Pathogen Free #1320, Lage, Germany) and tap water (ad libitum), under standardized housing and laboratory conditions (artificial 12-hour light / 12-hour dark cycle starting at 07.00 a.m.; temperature of 20 ± 2°C, relative humidity of 50 ± 15%; with at least 10 room air changes per hour). For 3 consecutive days, before inducing experimental seizures, the animals were subjected to the appropriate and skilled handling to familiarize the animals with the researchers’ contact. On the day of experiments, the animals were randomly assigned to experimental groups and maintained in small mouse experimental cages (8 mice per cage). Each animal was used only once and all experiments were carried out between 08.00 a.m. and 03.00 p.m. Each experimental group comprises 8 mice. All experiments involving animals complied with the ARRIVE guidelines and were conducted in strict accordance with the recommendations in the Guide for the Care and Use of Laboratory Animals of the National Institutes of Health and the EU Directive 2010/63/EU for animal experiments. The protocol using the animals was approved by the Local Ethics Committee for Animal Experimentation at the University of Life Sciences in Lublin, Poland (Protocol Number: 92/2018). Total number of mice used in this study was 416 (i.e., 17 groups per 8 mice in the 6-Hz corneal stimulation-induced seizure model [136 mice] for 5 antiepileptic drugs administered separately and 35 groups per 8 mice in the 6-Hz corneal stimulation-induced seizure model [280 mice] for 10 various two-drug combinations). All efforts were made to minimize animal suffering and to use only the number of animals necessary to produce reliable scientific data according to the 3Rs rule [31–34]. Because analgesic and anesthetic drugs administered systemically produce anti-seizure effects and can pharmacokinetically affect and/or change concentrations of antiepileptic drugs in mice, we did not administer such drugs, except for a local ocular anesthetic drug (0.5% solution of tetracaine hydrochloride as eye drops), inserted at 5 min. before the corneal stimulation-induced seizures (for more information see the “6-Hz corneal stimulation-induced seizures” section). After finishing the experiments, the animals were placed in a special uncharged chamber and euthanasia was performed by means of carbon dioxide (CO_2_). The exposure of animals to CO_2_ was performed using a gradual-fill method with a displacement rate from 30% to 70% of the chamber volume per minute, as recommended elsewhere [35]. Because this displacement rate is critical to the humane application of CO_2_, the appropriate pressure-reducing regulators and flow meters were used to precisely dosing gases from concentrated CO_2_ gas cylinders. After the animals become unconscious, the flow rate of CO_2_ was increased to minimize the time to death and the CO_2_ flow was maintained for at least 1 minute after respiratory arrest, as recommended elsewhere [35]. The number of animals that underwent euthanasia at the same time was limited in the chamber to allow free flow of CO_2_ to each animal and allow the animals to turn around, as suggested earlier [36]. The euthanasia of animals was performed only by a certified in euthanasia employee of the Experimental Medicine Center of Medical University of Lublin, Poland.

### Drugs

Gabapentin (GBP, Parke-Davis, Berlin, Germany), lacosamide (LCM, UCB Pharma, Brussels, Belgium), levetiracetam (LEV, UCB Pharma, Braine-l’Alleud, Belgium), pregabalin (PGB, Pfizer Ltd., Sandwich, Kent, UK), and retigabine (RTG, GlaxoSmithKline, Brentford, UK) were suspended in a 1% aqueous solution of Tween 80 (Sigma-Aldrich, Poznan, Poland). The 1% aqueous solution of Tween 80 (polysorbate 80) is a standard solvent used in pharmacological in vivo studies, especially, for lipophilic drugs that do not directly dissolve in water. Tween 80 changes lipophilicity of the antiepileptic drugs allowing for preparation of the drugs for i.p. injections [37]. It is widely accepted that the concentration of Tween 80 used in this study (1% aqueous solution) is not harmful for animals [38]. All the antiepileptic drugs were injected intraperitoneally (i.p.), in a volume of 5 ml/kg body weight, as follows: GBP and PGB– 120 min, LEV– 60 min, LCM– 30 min, and RTG– 15 min, before initiation of 6-Hz corneal stimulation-induced seizures, as recommended elsewhere [24, 26, 39–42]. All experiments were conducted in a blinded manner.

### 6-Hz corneal stimulation-induced seizures

Seizure activity in mice was evoked by current (32 mA, 6 Hz, 0.2 ms rectangular pulse width, 3 s duration) generated by an S48 Square Pulse Stimulator and CCU1 Constant Current Unit (Grass Technologies, West Warwick, RI, USA). Each mouse received a drop of ocular anesthetic (0.5% solution of tetracaine hydrochloride) to each eye at 5 min. before corneal stimulation of seizures. After corneal stimulation, each animal was immediately placed separately in a Plexiglas cage (25 × 15 × 10 cm) for the observation of seizure activity, as described previously [41, 43–45]. The seizure activity evoked by the 6-Hz corneal stimulation produced in each untreated mouse a “stunned” posture accompanied with rearing and automatic movements that are classified as convulsive and non-convulsive components of psychomotor seizures (including, immobility or stun, jaw and forelimb clonus, twitching of the vibrissae, and an elevated tail or Straub-tail), which lasted from 60 to 120 s [29, 46–48]. In this model, the mouse presenting either motor seizures or stereotypic movements accompanied with a “stunned posture” that lasted up to 120 s was classified as non-protected from seizures [41, 43–45]. In contrast, the protection from 6-Hz corneal stimulation-induced seizures was observed if the mouse resumed its normal exploratory behavior within 20 s after stimulation. To correctly classify the animal as protected or not, each mouse was observed for 40 s for the absence or presence of either convulsive or non-convulsive signs of psychomotor seizures, as recommended elsewhere [29, 46–48]. After finishing the observation of 8 mice in the respective group, the animals underwent euthanasia by CO_2_ narcosis. To determine median effective doses (ED_50_ values) for the antiepileptic drugs when administered alone, the drugs were administered systemically (i.p.) in the following doses: GBP– 50, 75, 100 mg/kg; LCM– 3, 5, 10, 15 mg/kg; LEV– 10, 15, 20 mg/kg; PGB– 15, 25, 50 mg/kg; and RTG– 20, 30, 35, 40 mg/kg (S1 Table). Total number of mice used for calculation of ED_50_ values for 5 antiepileptic drugs when administered alone was 136. Subsequently, to determine median effective doses for the two-drug mixtures (ED_50 mix_ values) for 10 various antiepileptic drug combinations, at least 3–5 mixtures of two drugs (at the fixed drug dose ratio combination of 1:1) were administered systemically (i.p.). Total number of mice used for calculation of ED_50 mix_ values for 10 various antiepileptic drug combinations was 280.

### Isobolographic analysis of interactions

To determine median effective doses (ED_50_ values ± S.E.M.) for the antiepileptic drugs, when administered alone, in the 6-Hz corneal stimulation-induced seizure model, a computer assisted log-probit linear regression analysis was used, as suggested earlier [49]. To isobolographically analyze the experimentally-derived data, the test for parallelism of dose-response relationship lines of the studied antiepileptic drugs (when injected alone) was used, as recommended earlier [45, 50–56]. The interactions for 10 various two-drug combinations against 6 Hz-corneal stimulation-induced seizures were analyzed isobolographically, as described earlier [22, 57–60]. This is the reason that the median effective additive doses (ED_50 add_ values ± S.E.M.) for 10 various two-drug mixtures, which theoretically protected 50% of the tested mice against 6-Hz corneal stimulation-induced seizures, were calculated from the ED_50_ values for the antiepileptic drugs administered alone, as described earlier [22, 58, 60]. Of note, proportions of two antiepileptic drugs in each mixture were calculated only for one fixed drug dose ratio combination of 1:1, as recommended earlier [15, 17, 23, 27, 53, 54, 61–66], and the respective mixtures were administered to animals. To determine the experimentally-derived median effective doses (ED_50 exp_ values ± S.E.M.) for the two-drug mixtures of antiepileptic drugs at the fixed-ratio of 1:1 in the 6-Hz corneal stimulation-induced seizure model, a log-probit linear regression analysis was used, as suggested earlier [49]. The isobolographic “additivity” in terms of the anticonvulsant effect produced by two-drug mixtures was defined in this study as the effect that was equal or almost equal to the sum of separate effects exerted by particular antiepileptic drugs comprised the mixture. Analogously, the isobolographic “supra-additivity” (synergy) was observed if the two-drug mixture exerted the anticonvulsant effect that was greater than the sum of the separate effects produced by the antiepileptic drugs combined together in mixtures. Both, the additivity and supra-additivity definitions are based on mass-action law [67–74]. Details concerning the isobolographic concepts and all required equations explaining the calculation of S.E.M. for ED_50 add_ values have been published earlier [60, 75–77]. Finally, to visualize all the types of interactions occurring between 5 antiepileptic drugs in the 6-Hz corneal stimulation-induced seizure model, we used a polygonogram, as recommended earlier [70, 78].

### Grip-strength test

The effects of 10 various antiepileptic drug combinations on skeletal muscular strength in mice were quantified by means of the grip-strength test, as recommended elsewhere [79, 80]. In this test, the mice received the respective drug mixture at the respective pretreatment times, and just before the 6-Hz corneal stimulation-induced seizures, the animals were subjected to the measurement of their skeletal muscular strength, as described earlier [81–83]. Briefly, each mouse was lifted by its tail and the mouse had to grip the steel grid connected to the electronic dynamometer with its forepaws. The maximal force of which each mouse released the grid with its forepaws was measured. Skeletal muscular strength in mice was expressed in newton (N) as means (± S.E.M. of 8 mice), for particular mixtures of two antiepileptic drugs in 10 various combinations. Since the grip-strength test was conducted on the same animals as those subjected to the 6-Hz corneal stimulation-induced seizures, there were no additional groups of mice tested in the grip-strength test.

### Statistical analysis

The ED_50_ values for antiepileptic drugs in the 6-Hz corneal stimulation-induced seizure model were calculated by log-probit analysis [49]. The experimentally-derived ED_50 exp_ values (± S.E.M.) were statistically compared with their respective additively calculated ED_50 add_ values (± S.E.M.) by the use of the unpaired Student’s *t*-test. Both, effect size and power for each tested combination of two antiepileptic drugs were computed by a “Compromise power analysis” in G*Power software (version 3.1.9.7 for Windows), as recommended elsewhere [33, 84, 85]. The results from the grip-strength test were statistically compared by the use of the one-way ANOVA. Differences among values were considered significant if *P*<0.05 by means of a GraphPad Prism software (version 7.0 for Windows; GraphPad Software, San Diego, CA, USA).

## Results

### Anticonvulsant effect of various antiepileptic drugs administered alone in the 6-Hz corneal stimulation-induced seizure model in mice

**Fig 1:**
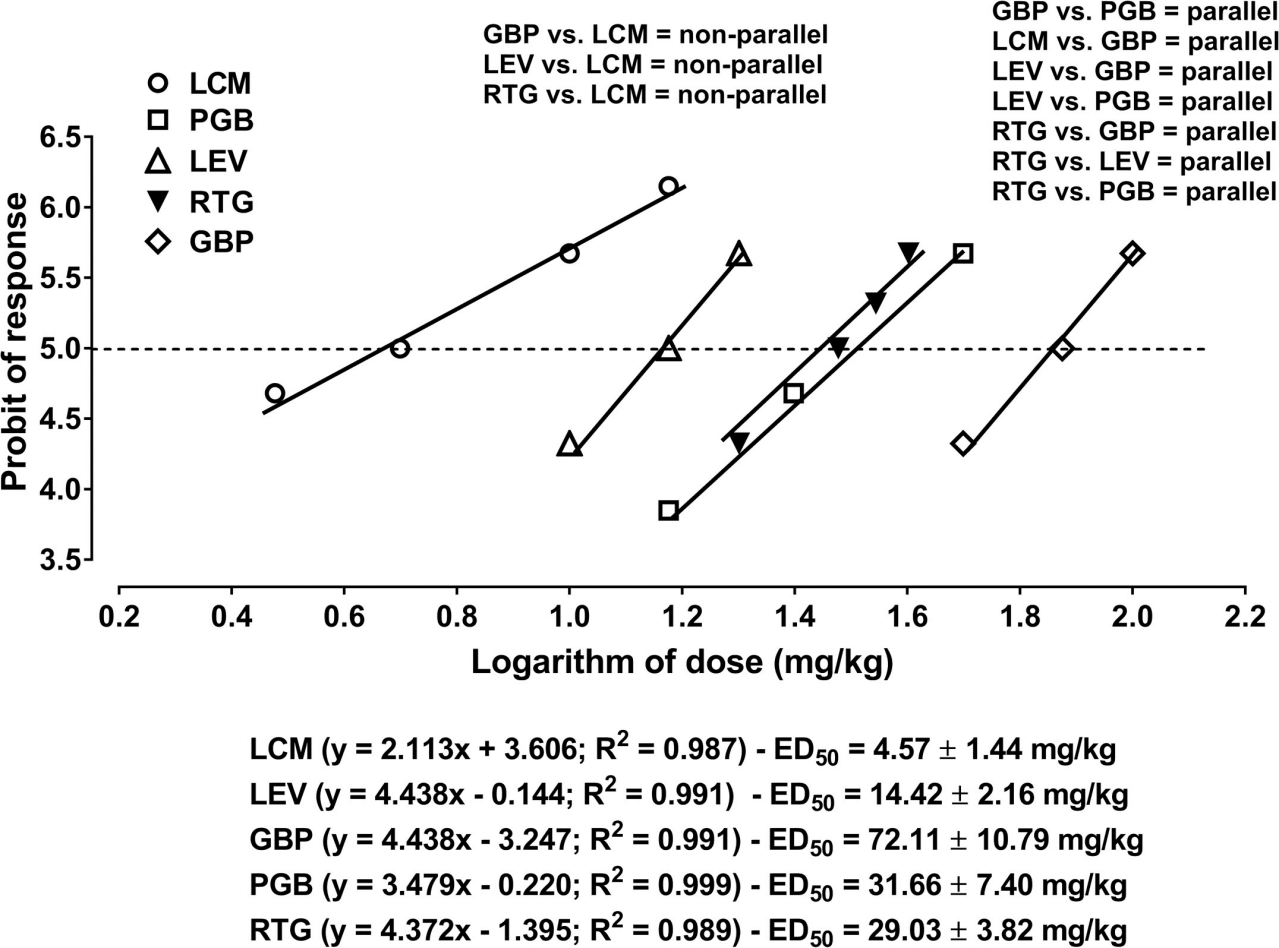
Anticonvulsant effects of gabapentin (GBP), lacosamide (LCM), levetiracetam (LEV), pregabalin (PGB) and retigabine (RTG) in the 6-Hz corneal stimulation-induced seizure model in mice. Doses of the antiepileptic drugs were transformed to logarithm to the base 10 and the protection of animals from 6-Hz corneal stimulation-induced seizures was transformed to probits, according to the log-probit method. Dose-response effects of the studied antiepileptic drugs were linearly related that allowed for calculating the ED_50_ values ± S.E.M. for all the tested drugs in the 6-Hz corneal stimulation-induced seizure model. Each data point corresponds to probit of mice protected (n = 8 mice/data point) from the 6-Hz corneal stimulation-induced seizures at a given logarithm of dose (in mg/kg). Intersections with the dashed line at 5 probit (50% effect) reflect approximate ED_50_ values of GBP, LCM, LEV, PGB and RTG, when administered alone. Test for parallelism of dose-response relationship lines for two antiepileptic drugs in the selected combinations was performed as recommended elsewhere [49].

Gabapentin, lacosamide, levetiracetam, pregabalin and retigabine when administered separately protected the mice from seizures evoked by the 6-Hz corneal stimulation (Fig 1, S1 Table). Linear equations for 5 antiepileptic drugs (when administered separately) presenting their dose-response relationships along with the experimentally derived ED_50_ values from the 6 Hz-corneal stimulation-induced seizure model in mice were presented on Fig 1. For 10 various possible two-drug combinations of antiepileptic drugs, it was found that only LCM had its dose-response relationship line non-parallel to those of GBP, LEV and RTG (Fig 1, S2 Table). In contrast, the other antiepileptic drugs in combination had their dose-response relationship lines collateral to each other (Fig 1, S2 Table).

### Anticonvulsant effects of 7 various two-drug combinations of antiepileptic drugs possessing their dose-response relationship lines collateral in the 6-Hz corneal stimulation-induced seizure model in mice

**Fig 2:**
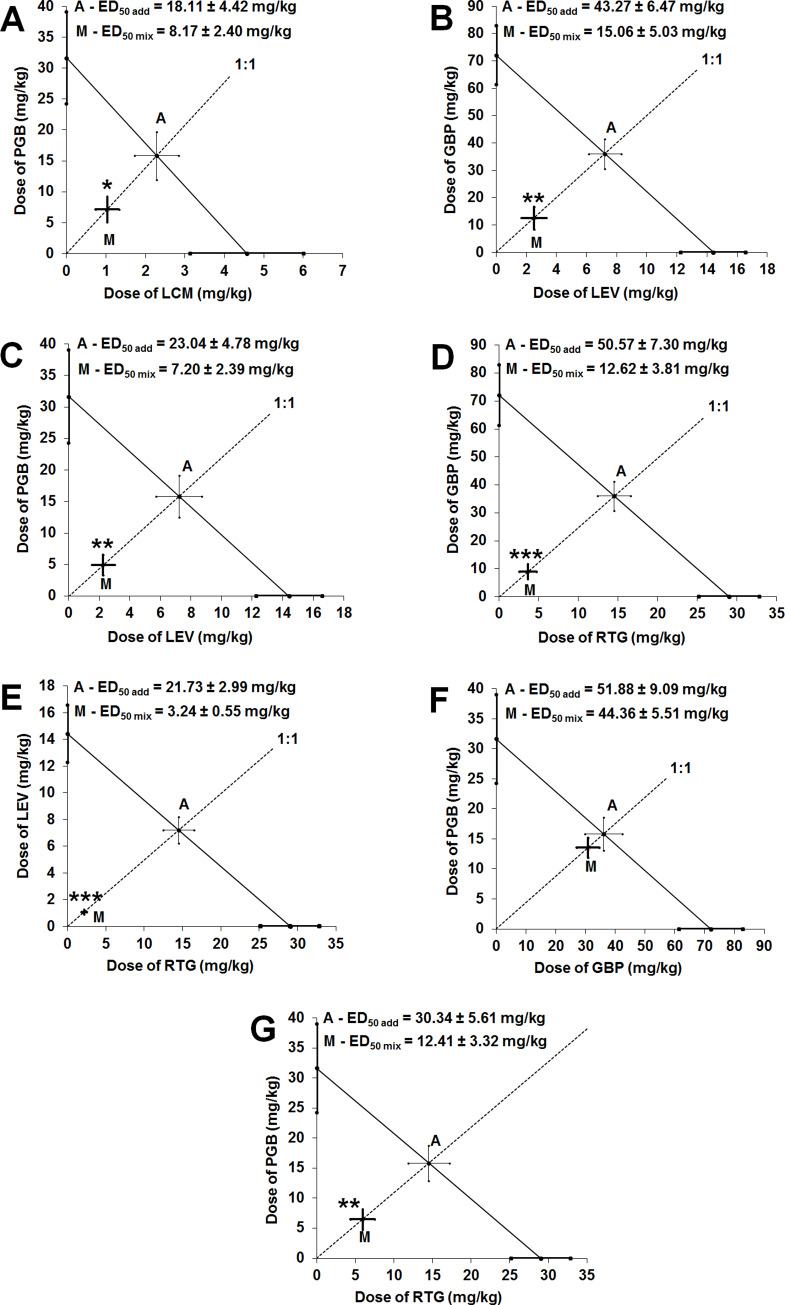
Isobolograms presenting interactions between gabapentin (GBP), lacosamide (LCM), levetiracetam (LEV), pregabalin (PGB) and retigabine (RTG), whose dose-response relationship lines were mutually collateral one another, in the 6 Hz-corneal stimulation-induced seizure model in mice. Isobolograms for various antiepileptic drugs combinations [PGB+LCM (A), GBP+LEV (B), PGB+LEV (C), GBP+RTG (D), LEV+RTG (E), PGB+GBP (F) and PGB+RTG (G)]. The ED_50_ of antiepileptic drugs when used alone (with S.E.M. as the error bars) are placed on the abscissa and ordinate of the Cartesian plot system. On each graph, the line connecting the ED_50_ values on both, X and Y axes illustrates the line of additivity. The point A reflects the ED_50 add_ value (with S.E.M. as the error bars) for the two-drug mixture that theoretically exerts additive interaction. The point M illustrates the ED_50 exp_ value (with S.E.M. as the error bars) for the two-drug combination that experimentally suppresses seizures in 50% of the animals tested. The dotted line crossing the points A and M illustrates the fixed drug dose ratio combination of 1:1. If the point M is placed significantly below the point A, the observed interaction is synergistic (unpaired Student’s t-test). *P<0.05, **P<0.01 and ***P<0.001 vs. the respective ED_50 add_ value. If the point M is placed close to the point A, the observed interaction is additive (unpaired Student’s t-test).

Type I isobolographic analysis for collateral dose-response relationship lines revealed that the two-drug combinations of PGB+LCM, GBP+LEV, PGB+LEV, GBP+RTG, LEV+RTG, and PGB+RTG at the fixed-ratio of 1:1 produced synergistic interaction in the 6-Hz corneal stimulation-induced seizure model in mice (Table 1; Fig 2A–2G; S3 Table). Statistical analysis with unpaired Student’s t-test with Welch’s correction confirmed that the experimentally determined ED_50 exp_ values considerably differed from their respective theoretically calculated additive ED_50 add_ values (Table 1; Fig 2A–2G). Only, the combination of PGB+GBP at the fixed-ratio of 1:1 exerted additive interaction in this seizure model in mice because statistical analysis of data revealed no significance between the respective ED_50 exp_ and ED_50 add_ values (Table 1; Fig 2F). Both, effect size and power for the tested combinations of antiepileptic drugs were computed by means of the “Compromise power analysis” (S4 Table).

**Table 1:** Type I isobolographic analysis of interactions for collateral dose-response relationship lines between antiepileptic drugs (at the fixed drug dose ratio of 1:1) in the 6-Hz corneal stimulation-induced seizure model in mice.

Drug combination	ED_50 exp_ (mg/kg)	*n*_exp_	ED_50 add_ (mg/kg)	*n*_add_	I-index	Types of interaction
**PGB + LCM**	8.17 ± 2.40 *	24	18.11 ± 4.42	36	0.45	synergy
**GBP + LEV**	15.06 ± 5.03 **	16	43.27 ± 6.47	44	0.35	synergy
**PGB + LEV**	7.20 ± 2.39 **	16	23.04 ± 4.78	36	0.31	synergy
**GBP + RTG**	12.62 ± 3.81 ***	8	50.57 ± 7.30	52	0.25	synergy
**LEV + RTG**	3.24 ± 0.55 ***	16	21.73 ± 2.99	52	0.15	synergy
**PGB + GBP**	44.36 ± 5.51	16	51.88 ± 9.09	36	0.86	additivity
**PGB + RTG**	12.41 ± 3.32 **	16	30.34 ± 5.61	44	0.41	synergy

The experimentally-derived median effective doses (ED_50 exp_ values in mg/kg ± S.E.M.) for two-drug mixtures were statistically compared to their respective theoretically calculated additive median effective doses (ED_50 add_ values in mg/kg ± S.E.M.) by the use of unpaired Student’s t-test with Welch’s correction as recommended elsewhere [60, 86–88]. *n*_exp_−total number of animals used at those doses whose expected antiseizure effects ranged between 16% and 84% (i.e., 4 and 6 probits) for the experimental mixture; *n*_add_−total number of animals calculated for the additive mixture of the drugs examined (*n*_add_ = *n*__antiepileptic drug_1_ + *n*__antiepileptic drug_2_−4); I-index–interaction index as a ratio of ED_50 exp_ and ED_50 add_ values

*P<0.05

**P<0.01

***P<0.001 vs. the respective ED_50 add_ value.

### Anticonvulsant effects of 3 various two-drug combinations of antiepileptic drugs having their dose-response relationship lines non-parallel in the 6-Hz corneal stimulation-induced seizure model in mice

**Fig 3:**
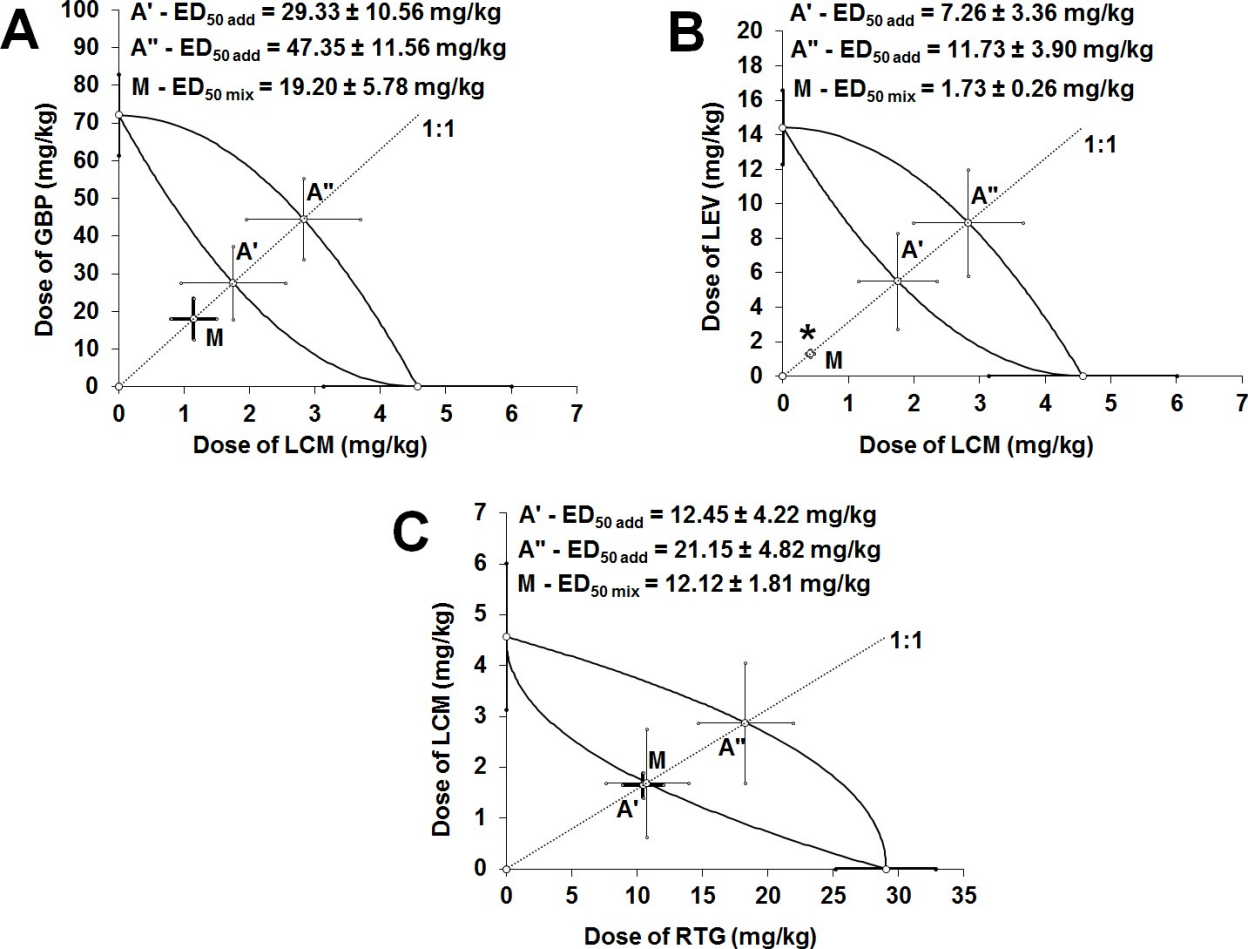
Isobolograms presenting interactions between gabapentin (GBP), lacosamide (LCM), levetiracetam (LEV), and retigabine (RTG), which had their dose-response relationship lines non-parallel, in the 6 Hz-corneal stimulation-induced seizure model in mice. Isobolograms for various antiepileptic drugs combinations [GBP+LCM (A), LEV+LCM (B) and RTG+LCM (C)]. The ED_50_ of antiepileptic drugs when used alone (with S.E.M. as the error bars) are placed on the abscissa and ordinate of the Cartesian plot system. On each graph, the lines connecting the ED_50_ values on both, X and Y axes represent lower and upper lines of additivity with points A’ and A”, reflecting the ED_50 add_ values (with S.E.M. as the error bars) for the two-drug mixture that theoretically exerts additive interaction for lower and upper lines, respectively. The point M illustrates the ED_50 exp_ value (with S.E.M. as the error bars) for the two-drug combination that experimentally suppresses 6-Hz corneal stimulation-induced seizures in 50% of the animals tested. The dotted line crossing the points A’, A” and M illustrates the fixed drug dose ratio combination of 1:1. If the point M is placed significantly below the area of additivity (shaped by both, lower and upper lines of additivity), the observed interaction is synergistic (unpaired Student’s t-test). *P<0.05 vs. the respective ED_50 add_ value. If the point M is placed close to the point A’, the interaction is additive in nature.

With type I isobolographic analysis for non-parallel dose-response relationship lines only one combination of LEV+LCM at the fixed-ratio of 1:1 exerted synergistic interaction in the 6-Hz corneal stimulation-induced seizure model in mice (Table 2; Fig 3B; S3 Table). In this case, the unpaired Student’s t-test with Welch’s correction revealed that the ED_50 exp_ value significantly differed from the respective ED_50 add_ values (P<0.05; Table 2; Fig 3B). In contrast, the combinations of GBP+LCM and RTG+LCM at the fixed-ratio of 1:1 produced additive interaction in the 6-Hz corneal stimulation-induced seizure model in mice (Table 2; Fig 3A and 3C). No statistical significance was reported for the two-drug combinations of GBP+LCM and RTG+LCM confirming additivity for these combinations in the 6-Hz corneal stimulation-induced seizure model in mice (Table 2; Fig 3A and 3C). Both, effect size and power for the tested combinations of antiepileptic drugs were computed by means of the “Compromise power analysis” (S4 Table).

**Table 2:** Type I isobolographic analysis of interactions for non-parallel dose-response relationship lines between antiepileptic drugs (at the fixed drug dose ratio of 1:1) in the 6-Hz corneal stimulation-induced seizure model in mice.

Drug combination	ED_50 exp_ (mg/kg)	*n*_exp_	*L*-ED_50 add_ (mg/kg)	*n*_add_	*U*-ED_50 add_ (mg/kg)	*n*_add_	I-index	Types of interaction
**GBP + LCM**	19.20 ± 5.78	8	29.33 ± 10.56	44	47.35 ± 11.56	44	0.65	additivity
**LEV + LCM**	1.73 ± 0.26 *	24	7.26 ± 3.36	44	11.73 ± 3.90	44	0.24	synergy
**RTG + LCM**	12.12 ± 1.81	24	12.45 ± 4.22	52	21.15 ± 4.82	52	0.97	additivity

The experimentally-derived median effective doses (ED_50 exp_ values in mg/kg ± S.E.M.) for two-drug mixtures were statistically compared to their respective theoretically calculated additive median effective doses (ED_50 add_ values in mg/kg ± S.E.M.) by the use of unpaired Student’s t-test with Welch’s correction as recommended elsewhere [60, 86–88]. *n*_exp_−total number of animals used at those doses whose expected antiseizure effects ranged between 16% and 84% (i.e., 4 and 6 probits) for the experimental mixture; *n*_add_−total number of animals calculated for the additive mixture of the drugs examined (*n*_add_ = *n*__antiepileptic drug_1_ + *n*__antiepileptic drug_2_−4)

*L*-ED_50 add_ value calculated from the equation for the lower line of additivity; *U*-ED_50 add_ value calculated from the equation for the upper line of additivity; I-index–interaction index as a ratio of ED_50 exp_ and *L*-ED_50 add_ values

*P<0.05 vs. the respective ED_50 add_ value.

### Polygonogram and interaction indices for 10 various two-drug combinations of antiepileptic drugs

**Fig 4:**
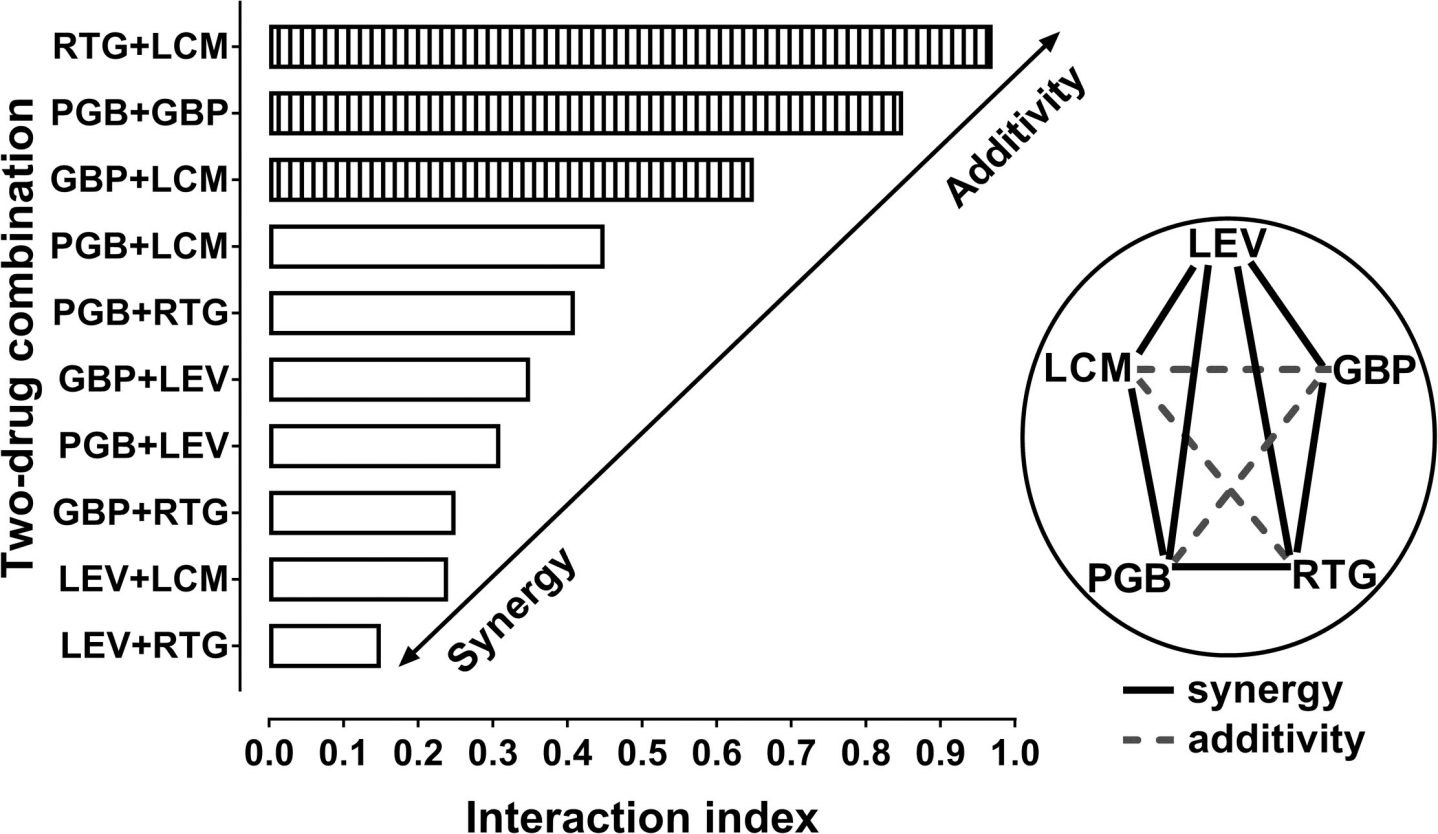
Polygonogram and interaction indices for two-drug combinations illustrating both synergistic and additive interactions among selected antiepileptic drugs in the 6-Hz corneal stimulation-induced seizure model in mice. Polygonogram for 5 antiepileptic drugs with different molecular mechanisms of actions. Levetiracetam (LEV), lacosamide (LCM), pregabalin (PGB), retigabine (RTG) and gabapentin (GBP), were combined together and the two-drug mixtures (at the fixed-ratio of 1:1) underwent isobolographic evaluation in the mouse 6-Hz corneal stimulation-induced seizure model. Solid lines indicate synergism between the investigated antiepileptic drugs, whereas the dashed lines indicate additive interaction. Interaction indices for the studied two-drug combinations of antiepileptic drugs are presented as vertical columns. White columns illustrate synergistic interaction, whereas stripped columns present additive interactions among the tested drugs.

The calculated interaction indices (being a measure of strength of interaction between antiepileptic drugs) ranged from 0.15 for the combination of LEV+RTG to 0.97 for the combination of RTG+LCM (Fig 4). The interaction indices for 7 two-drug combinations of LEV+RTG, LEV+LCM, GBP+RTG, PGB+LEV, GBP+LEV, PGB+RTG, PGB+LCM were lower than 0.6 indicating synergistic interaction between the studied antiepileptic drugs. On the contrary, the interaction indices for 3 two-drug combinations of GBP+LCM, PGB+GBP and RTG+LCM were higher than 0.6 illustrating additive interaction between the studied antiepileptic drugs (Fig 4).

### Effects of 10 various two-drug combinations of antiepileptic drugs on skeletal muscular strength in mice

One-way ANOVA revealed that 10 two-drug combinations of antiepileptic drugs (at doses corresponding to the halves of ED_50_ values of the antiepileptic drugs from the 6-Hz corneal stimulation-induced seizure model) did not alter skeletal muscular strength in mice challenged with the grip strength test, which was performed before the induction of seizures in experimental animals (Table 3).

**Table 3:** Effects of 10 various combinations of gabapentin (GBP), lacosamide (LCM), levetiracetam (LEV), pregabalin (PGB) and retigabine (RTG) on skeletal muscular strength in the grip-strength test in mice.

Treatment (mg/kg)	Grip-strength (N)
**Vehicle + vehicle**	0.901 ± 0.050
**PGB (15.83) + LCM (2.28)**	0.887 ± 0.055
**GBP (36.06) + LEV (7.21)**	0.892 ± 0.058
**PGB (15.83) + LEV (7.21)**	0.896 ± 0.049
**GBP (36.06) + RTG (14.52)**	0.902 ± 0.050
**LEV (7.21) + RTG (14.52)**	0.898 ± 0.051
**PGB (15.83) + GBP (36.06)**	0.892 ± 0.057
**PGB (15.83) + RTG (14.52)**	0.907 ± 0.049
**GBP (36.06) + LCM (2.28)**	0.903 ± 0.052
**LEV (7.21) + LCM (2.28)**	0.892 ± 0.055
**RTG (14.52) + LCM (2.28)**	0.905 ± 0.057

Results are mean strengths (in newtons ± S.E.M. of 8 animals per group) from the grip-strength test, assessing skeletal muscular strength in mice.

## Discussion

The two-drug combinations of various second-generation antiepileptic drugs tested in the current study exerted mostly synergistic interaction in the 6-Hz corneal stimulation-induced seizure model in mice. More specifically, it was found that the combinations of LEV with LCM, GBP, PGB and RTG produced synergistic interaction. Combinations of RTG with LEV, PGB and GBP were synergistic, but RTG combined with LCM exerted only additive interaction in the mouse 6-Hz corneal stimulation seizure model. In case of PGB, the drug synergistically interacted with LCM, LEV and RTG in terms of seizure suppression in experimental animals, and only the combination of PGB with GBP was additive in the 6-Hz corneal stimulation-induced seizure model. GBP when combined with RTG and LEV exerted synergistic interaction, whereas the combinations of GBP with PGB and LCM were additive. LCM exerted both, synergistic interaction with LEV and PGB, and additive interaction with GBP and RTG in the 6-Hz corneal stimulation-induced seizure model. Of note, we investigated in this study all possible interactions, which may occur between 5 various antiepileptic drugs in two-drug combinations (Fig 4). Considering the above-mentioned results, it seems that the combinations containing LEV should be preferentially implied to clinical practice because all of the studied combinations with LEV produced synergistic interactions in mice subjected to the 6-Hz corneal stimulation-induced seizure model. To display all types of interactions that occurred between 5 antiepileptic drugs, a polygonogram was used, which is a simple graphical illustration of types of interactions between drugs. Primarily, it has been introduced to illustrate interactions observed between anti-cancer (anti-proliferative) drugs. However, a simplicity of drawing the polygonogram allowed us to incorporate this graph to experimental epileptology in order to tangibly visualize types of interactions between antiepileptic drugs in terms of seizure suppression in experimental animals.

To isobolographically characterize types of interactions between the second-generation antiepileptic drugs, the test of parallelism of dose-response relationship lines was used before testing experimental mixtures of two antiepileptic drugs in the 6 Hz corneal stimulation seizure model. To unify and compare the results from both, parallel and non-parallel isobolographic analysis methods, only one fixed drug dose ratio combination of 1:1 was chosen to be tested, in which doses of particular antiepileptic drugs in the mixture were equi-effective. In other words, the drug mixtures for various antiepileptic drug combinations exerted the same (50%) effects in animals subjected to the 6 Hz corneal stimulation-induced seizure model.

It should be stressed that in the 6 Hz corneal stimulation-induced seizure model we used a current intensity of 32 mA, which was associated with full efficacy of LEV and other tested antiepileptic drugs in suppression of limbic (psychomotor) seizures in mice. Since all of the tested antiepileptic drugs produced the definite antiseizure effects in the 6-Hz corneal stimulation-induced seizure model, it was possible to correctly classify interactions between the second-generation antiepileptic drugs by means of the type I isobolographic analysis of interaction, as recommended elsewhere [22, 51–53, 88–90].

The main clinical question arises whether we still need combinations of antiepileptic drugs to treat epileptic patients with refractory seizure attacks. Combinations of antiepileptic drugs are usually associated with various interactions whose nature may be pharmacokinetic, pharmacodynamic or mixed [10, 91]. From a clinical perspective, the most desired combination of antiepileptic drugs is that exerting synergy between drugs in relation to their anticonvulsant effects [12, 92]. Clinicians usually expect that some antiepileptic drugs generate synergistic interactions when the drug are combined together, but some combinations may also be additive or even antagonistic. It should be stressed that antagonistic interactions might not be properly recognized by clinicians during pharmacotherapy of epilepsy, because the clinical manifestation of antagonistic interaction is usually associated with lack of control on seizures in patients. Thus, patients receiving two antiepileptic drugs in combination that produced antagonistic interaction have still seizures. From clinical standpoint, any patients who have still seizures (even if they result from antagonistic interactions between antiepileptic drugs) may be considered to have refractory epilepsy and they need some efficacious treatment options. In such cases, the ineffective antiepileptic drugs are usually replaced with other more effective antiepileptic drugs and this, probably, is the main reason that antagonistic interactions between antiepileptic drugs are not recognized by physicians in their clinical practice.

During isobolographic experimental assessment of interaction between 2 drugs, one of the principal stages is to evaluate the ED_50_ values for the drugs when they are administered alone. This step is always the same for various antiepileptic drug combinations tested isobolographically. In other words, before evaluating the ED_50 exp_ values for various two-drug mixtures, researchers are obliged to calculate the theoretically additive ED_50 add_ values for the mixtures, which can be considered as additive, or more precisely, researchers calculate doses of two-drug mixtures, whose effects are additive from the theoretical viewpoint. This calculation is a standard procedure in isobolographic study and after determining the ED_50_ values for the 5 antiepileptic drugs used alone, we could use these values to analyze 10 various possible antiepileptic drug combinations. Thus, a considerable reduction of number of experimental animals was achieved, which was in accordance with the 3Rs rules (Reduction, Replacement and Refinement) and ARRIVE guidelines during the use of laboratory animals [32]. Experimental evaluation of ED_50 exp_ values for 10 different antiepileptic drug combinations allowed us to provide evidence on types of interactions occurring between 5 antiepileptic drugs in all possible constellations of combinations in the 6-Hz corneal stimulation-induced seizure model.

In this study we determined the ED_50_ and ED_50 exp_ values, based on number of animals protected from 6 Hz corneal stimulation-induced seizures. Because all the studied antiepileptic drugs exerted clear-cut antiseizure effects, the doses of the drugs were set up so as to readily determine the ED_50_ and ED_50 exp_ values. In this study we analyzed the dose-dependent response of the antiepileptic drugs and their mixtures to their anticonvulsant protective effects and the most crucial was the assessment of drugs’ ability to suppress seizures. Both, seizure severity and seizure duration in animals subjected to the 6 Hz corneal stimulation-induced seizures were not examined in this study because there was no control (drug-naïve mice) group to which the respective antiepileptic drugs could be derived and compared. The analysis of seizure severity and seizure duration is usually performed for drugs administered in constant doses, when it is possible to compare the effects produced by the investigated substances or drugs to those observed in drug-naïve animals [47, 93, 94]. This was the reason that neither “responder” nor “non-responder” subgroups of animals were defined in this study. Besides, there was no need for such differentiation of animals because of diverse methodological approaches used in this study and those by other authors [47, 93, 94].

Assessment of interaction for two-drug mixtures of antiepileptic drugs in preclinical studies contributes to the creation of the list of favorable combinations that would be recommended to clinical practice, when selecting antiepileptic drugs for epilepsy patients [7–9]. Almost two decades ago, it has been clinically documented that some antiepileptic drugs in combination were better than others and their clinical application was recommended by physicians. At present, only few antiepileptic drug combinations are clinically recommended by physicians because of their high clinical efficacy in epilepsy patients [7–9]. Although a direct translation of preclinically favorable antiepileptic drug combinations to clinical settings is often impossible (because of different doses of used drugs), a concept of implying only synergistic interactions of antiepileptic drugs to the patients is worthy of being introduced to clinical practice. The process of transition from animals to humans is difficult, but the characteristics of interaction remain the same in both, animals and humans. This is the reason to recommend to clinical practice only those antiepileptic drug combinations, which were preclinically verified as synergistic with the isobolographic method.

Previously, it has been found that some antiepileptic drugs in combination produced both, additive and synergistic interactions in the 6-Hz corneal stimulation-induced seizure model. More specifically, LEV combined with clonazepam, oxcarbazepine, tiagabine, and valproate produced additive interaction, while LEV in combination with phenobarbital exerted supra-additive (synergistic) interaction in mice subjected to the 6-Hz corneal stimulation-induced seizure model [45]. The combinations of LCM with phenytoin and valproate were additive, but the combinations of LCM with lamotrigine, tiagabine, GBP, carbamazepine and LEV occurred synergistic in the 6-Hz corneal stimulation-induced seizure model in mice [95]. However, an essential discrepancy can be observed between the results for the combination of LCM with GBP in our study and that found by other authors. For instance, in the study by Shandra et al., [95], the mixture of LCM and GBP produced synergistic interaction. Unfortunately, the isobolographic analysis performed earlier did not differentiate that the tested antiepileptic drugs possessed their dose-response relationship lines non-parallel to each other [95]. Obviously, the lack of parallelism between the antiepileptic drugs substantially modified the tested interaction because 2 ED_50 add_ values for lower and upper isoboles of additivity were calculated for non-parallel dose-response relationship lines for LCM and GBP in this study. This is the main reason contributing to the fact that our results, showing additivity for the combination of LCM with GBP, differ from those previously published by other authors, who have found synergistic interaction between LCM and GBP in the 6-Hz corneal stimulation-induced seizure model in mice. Furthermore, different doses of antiepileptic drugs tested in both experiments should explain at least in part the observed discrepancies in types of interactions between LCM and GBP in the mouse 6-Hz corneal stimulation-induced seizure model. For instance, the ED_50_ value for LCM was 10.1 (4.5–19.8) mg/kg and for GBP was 224.0 (108–428) mg/kg, respectively [95]. In contrast, in our study, the ED_50_ values for the antiepileptic drugs were substantially lower amounting to 4.57 (2.5–8.5) mg/kg for LCM and 72.11 (53.8–96.7) mg/kg for GBP, respectively.

Analysis of isobolographic interactions with polygonogram allowed for ascertaining that between GBP and PGB must exist any kind of difference in their anticonvulsant profiles in the 6-Hz corneal stimulation-induced seizure model in mice. More specifically, the interaction for the combination of LCM with GBP was additive, whereas that for LCM with PGB was synergistic in the current study. If the molecular mechanisms of action of PGB and GBP were identical in both drugs and related exclusively with the blockade of α2δ subunit of calcium channels [96–98], the interaction of GBP and PGB with LCM would be the same. On the contrary, the synergistic interaction between LCM and PGB suggests that the anticonvulsant efficacy of PGB was greater than that for GBP when combined with LCM, exerting additive interaction. Difference in the interaction types in mice receiving the mixture of LCM with GBP or PGB resulted probably from various molecular mechanisms of action of the tested antiepileptic drugs, but this hypothesis needs verification in further neuro-bio-molecular studies.

It is important to note that in this study we calculated the interaction index values (as a ratio of ED_50 exp_ and ED_50 add_ values) in order to assess the strength of interaction occurring between antiepileptic drugs. The most synergistic interaction was that between LEV and RTG, for which the interaction index value was the lowest one amounting to 0.15 (Fig 4). On the other hand, the most additive interaction was that between RTG and LCM, for which the interaction index was 0.97 (Fig 4). Of note, the interaction index values adequately describe the types of interactions occurring for drugs possessing their dose-response relationship lines parallel to one another [40, 72, 99]. In contrast, the interaction index values cannot precisely determine the strength of interaction between antiepileptic drugs whose dose-response relationship lines are not collateral [78, 99]. In such a situation, the additive area bounded by two lower and upper lines of additivity did not allow to precisely correlate one ED_50 exp_ value with two ED_50 add_ values and calculate the interaction index value. In this study, statistical analysis of data was based on comparison of the ED_50 exp_ with ED_50 add_ values by means of the unpaired Student's t-test with Welch's correction, as recommended elsewhere [77, 86, 88]. The calculation of interaction index values in this study was an additional method allowing for classification of the strength of interaction between antiepileptic drugs. Several years ago, the calculation of interaction index value was the unique method when classifying interactions as additive, synergistic or antagonistic [68, 72, 73, 100, 101]. With the advances in elaboration of type I isobolographic analysis of interaction for non-parallel dose-response relationship lines, the application of interaction index in experimental studies has been drastically limited. Today, only the statistical analysis of data with Student’s t-test remains the one acceptable and preferred method for classification of interactions between drugs [57].

In case of administration of RTG in this study, we are fully aware of the fact that the drug was not allowed anymore to treat the patients with epilepsy. Since RTG produced irreversible blue-gray discoloration of the skin, nails, sclera and conjunctiva in some epileptic patients, the clinical administration of RTG has been drastically limited and all clinical trials with RTG have been terminated [102, 103]. However, other drugs (i.e., XEN1101, KB-3061) with similar mechanisms of action related with activation of K+ channels, but devoid of any harmful adverse effects like RTG, could be used in epileptic patients to treat their seizures [104–107]. This was the reason to preclinically investigate RTG because the drug as a K+ channel activator can significantly contribute to the suppression of seizures if it could be combined with other antiepileptic drugs possessing various molecular mechanisms of action. Recently, a suggestion of combining antiepileptic drugs affecting simultaneously various targets in the epileptic brain, as a result of activation of various molecular mechanisms of action, has gained popularity not only among clinicians, but also among epileptologists [108].

The main limitation of this study is the acute administration of the antiepileptic drugs. Of note, in clinical conditions, the antiepileptic drugs are usually administered chronically and they can mutually influence their own metabolisms by inhibiting or enhancing liver enzymes responsible for antiepileptic drug degradation and elimination. During chronic administration, the antiepileptic drugs reach pharmacokinetic steady state and some of them undergo metabolic transformation to inactive compounds or to some active metabolites. This is the principal reason that their anticonvulsant effects may differ between acute and chronic administration. The main goal of this study was to determine the anticonvulsant effects of several two-drug mixtures injected singly (acutely) in the 6 Hz corneal stimulation-induced seizure model to select the most beneficial combinations of antiepileptic drugs. From a methodological point of view, it is possible to determine the anticonvulsant effects of the antiepileptic drugs administered chronically in the same seizure model. Analogously, it is possible to administer the antiepileptic drugs in mixture in high doses so as to determine their tolerability and adverse effect profiles, but such experiments are usually conducted in various behavioral animal models assessing untoward effects exerted by the antiepileptic drugs when injected in high doses [79, 109–112]. Of note, chronically administered antiepileptic drugs can change their own profiles due to the development of resistance and/or tolerance to the drugs [113, 114]. On the other hand, the chronic induction of 6 Hz corneal stimulation-induced seizures associated with repeated electric stimulations in experimental animals is responsible for progressive seizure aggravation in rodents [47, 93, 94]. Undoubtedly, the repeating seizure induction in mice makes that animals become refractory to the antiepileptic drugs. At present, no experiments investigating the antiepileptic drugs’ effectiveness were conducted isobolographically in in vivo studies based on repeated 6 Hz corneal stimulation-induced seizures and chronically administered drugs. Perhaps, more advanced studies will focus on these research problems in near future.

Another limitation in this study is related with low doses of the tested antiepileptic drugs. Due to synergistic interactions between antiepileptic drugs in the 6-Hz corneal stimulation-induced seizures model, doses of particular antiepileptic drugs used in combination were substantially low and thus, they could not evoke pharmacokinetic interaction among drugs. Additionally, low doses of antiepileptic drugs contribute to the reduction of side effects observed in case when the antiepileptic drugs are used separately, but in high doses. Besides, no symptoms of acute adverse effects were observed in animals subjected to the grip-strength test just before electrically evoked 6-Hz corneal stimulation seizures.

Assessment of skeletal muscular strength in mice that received various antiepileptic drug combinations at the fixed-ratio of 1:1, in doses corresponding to halves of the ED_50_ values of the tested antiepileptic drugs and just before the 6-Hz corneal stimulation-induced seizures, allowed us to ascertain that there was no impairment in muscular skeletal strength in mice, as compared to the control group. Since the muscular strength did not differ between groups of animals receiving various antiepileptic drug combinations, we can ascertain that doses of antiepileptic drugs were devoid of any adverse effects in animals. It should be stressed that by assessing skeletal muscular strength in mice we tried to evaluate behavioral manifestation of acute adverse effects produced by the antiepileptic drugs in combination. Generally, a risk of acute adverse effects occurrence increases along with a number of antiepileptic drugs combined together. In this study, we confirmed that all the studied combinations of antiepileptic drugs did not produce any impairment in muscular strength in mice that received antiepileptic drugs in doses corresponding to the halves of ED_50_ values from the 6-Hz corneal stimulation-induced seizures. Of note, the grip-strength test was performed before the 6-Hz corneal stimulation-induced seizures on the same animals, which allowed us not to use additional groups of animals in this study to investigate adverse effect potential of antiepileptic drugs in combination. Both, simplicity of testing and quick performance of the grip-strength test in animals did not disturb the evaluation of protective effects of the antiepileptic drugs in mixtures in the 6-Hz corneal stimulation-induced seizure test. Thus, we elaborated a new pattern for screening acute adverse effects in animals, without using additional animals. We enriched our experimental procedure when determining the anticonvulsant properties of the antiepileptic drugs in mixtures in the 6-Hz corneal stimulation-induced seizure test in mice by additional testing of the skeletal muscular strength in the mice prior to the induction of seizures. Generally, other behavioral tests evaluating acute adverse effects in animals including, the passive avoidance task and/or Y-, Plus-maze tests are time-consuming behavioral tests [28, 115, 116], which cannot be conducted before the 6-Hz corneal stimulation-induced seizures on the same animals. On the other hand, considering low doses of particular drugs and their mixtures used in the 6-Hz corneal stimulation-induced seizure test, impairment of motor coordination in the animals was not likely to occur. This was the reason not to conduct the chimney or rotarod tests on the same animals before the 6-Hz corneal stimulation-induced seizures. It is widely accepted that the median toxic doses (TD_50_ values) for the studied antiepileptic drugs, as determined in the chimney or rotarod tests were considerably higher than their corresponding ED_50_ values as determined in the 6 Hz corneal stimulation-induced seizures (for more detail see S5 Table).

The selection of the second-generation antiepileptic drugs investigated in the 6-Hz corneal stimulation model was not serendipitous, but minutely verified. The preferential selection of GBP, LCM, LEV, PGB and RTG investigated in the 6 Hz corneal stimulation seizure model was based not only on molecular mechanisms of action of the tested antiepileptic drugs, but primarily on their pharmacokinetic profiles. In other words, the safe pharmacokinetic profiles of antiepileptic drugs were the main criterion for selecting these antiepileptic drugs and test them in the 6-Hz corneal stimulation-induced seizure model. It can be highlighted that these antiepileptic drugs cannot mutually affect their pharmacokinetic parameters. It is well-known that LEV, PGB and GBP do not undergo metabolic transformation by the liver microsomal enzyme (CYP) system and they are mainly eliminated as unchanged drugs with urine [108]. In case of LCM, the drug undergoes demethylation by CYP3A4, CYP2C9 and CYP2C19 isoenzymes in the liver, whereas RTG undergoes N-acetylation and N-glucuronidation in the liver thereby UGT1A1, UGT1A3, UGT1A4 and UGT1A9 isoenzymes [108]. Thus, the risk of pharmacokinetic interaction between the tested antiepileptic drugs is low, especially, when combining antiepileptic drugs with various pharmacokinetic profiles that are completely different and the drugs are metabolized and eliminated through different pathways. Considering the above-mentioned facts we can suppose that the existence of any pharmacokinetic interactions between the studied second-generation antiepileptic drugs is unlikely. Besides, low doses of antiepileptic drugs in mixtures for various antiepileptic drug combination tested in the 6-Hz corneal stimulation-induced seizure model are not expected to produce pharmacokinetic interactions.

## Conclusions

Summing up, 7 two-drug combinations of LEV+RTG, LEV+LCM, GBP+RTG, PGB+LEV, GBP+LEV, PGB+RTG, PGB+LCM, producing synergistic interaction in the mouse 6-Hz corneal stimulation-induced seizure model can be recommended to clinical practice as favorable combinations. Three other combinations of GBP+LCM, GBP+PGB, and RTG+LCM that exerted additivity in animals can also be applied in patients with epilepsy, especially, if the beneficial effects resulting from their anticonvulsant protection from seizures greatly outweigh the risk of acute adverse effects occurrence. The results from this preclinical study can be translated to clinical conditions, however, doses of the respective drugs in combination need adjustment to patients and transfer from the mouse to human organism. Polygonogram illustrating types of interactions among all the studied antiepileptic drugs, can be very helpful for clinicians allowing for the choice of the drugs that synergistically suppressed seizures in animals. We recommend to visualize isobolographically-derived interactions among drugs by means of polygonogram along with interaction indices that characterize strength of the investigated combinations of the antiepileptic drugs.

## Supporting information

S1 Table Anticonvulsant activity of gabapentin (GBP), lacosamide (LCM), levetiracetam (LEV), pregabalin (PGB) and retigabine (RTG) when administered separately in the 6-Hz corneal stimulation-induced seizure model in mice. Doses of particular antiepileptic drugs used in the 6-Hz corneal stimulation-induced seizure model are placed in the first column. Results indicate numbers of animals protected from 6-Hz corneal stimulation-induced seizures per total number of animals in each experimental group.
(DOC)

S2 Table Anticonvulsant effects of gabapentin (GBP), lacosamide (LCM), levetiracetam (LEV), pregabalin (PGB) and retigabine (RTG) administered singly in the 6-Hz corneal stimulation-induced seizure model in mice. For each tested combination of two antiepileptic drugs results indicate median effective doses (ED_50_ values in mg/kg ± S.E.M.) of the studied antiepileptic drugs, when administered separately, in the 6-Hz corneal stimulation-induced seizure model in mice. Test for parallelism of dose-response relationship lines for two antiepileptic drugs in the selected combinations was performed according to Litchfield and Wilcoxon [49]. In this test, if the slope function ratio (S.R.) value is higher than the factor for slope function ratio (f ratio S.R.) value, the examined two lines are non-parallel to each other [49]. On the contrary, if the S.R. value is higher than the f ratio S.R. value, the studied two lines are collateral each other [49]. n—total number of mice used at those doses whose expected antiseizure effects ranged between 4 and 6 probits; CFP–(q and p) curve-fitting parameters; q/p—ratio of q and p values; S.R.–slope function ratio; f ratio S.R.–factor for slope function ratio. N.P.—not parallel; P.–parallel. All calculations necessary to confirm the parallelism of two antiepileptic drugs’ lines were presented in more detail elsewhere [15, 24, 75, 117].
(DOC)

S3 Table Anticonvulsant activity of 10 various two-drug combinations of gabapentin (GBP), lacosamide (LCM), levetiracetam (LEV), pregabalin (PGB) and retigabine (RTG) administered at the fixed-ratio of 1:1 in the 6-Hz corneal stimulation-induced seizure model in mice. Doses of particular AEDs in combination tested in the 6-Hz corneal stimulation-induced seizure model are placed in the first column. Results indicate numbers of animals protected from 6-Hz corneal stimulation-induced seizures per total number of animals in each experimental group.
(DOC)

S4 Table Power analysis for the tested antiepileptic drug combinations in the 6-Hz corneal stimulation-induced seizure model in mice. Both, effect size and power for each tested combination of two antiepileptic drugs were computed by a “Compromise power analysis” in G*Power software (version 3.1.9.7 for Windows–freely available) that allowed the respective calculations based on ED_50_, S.D. values and numbers of animals tested, as recommended elsewhere [84, 85]. In animal studies, the power equal to and higher than 0.8 (at the significance level of 0.05) is sufficiently accepted. S.D.–standard deviation; N–total number of animals used in the 6-Hz corneal stimulation-induced seizure test to calculate ED_50 exp_ values; n–total number of animals used to calculate the ED_50 add_ values (i.e., n = N__antiepileptic drug 1_ + N__antiepileptic drug 2_−4); d.f.–degree of freedom; t–critical t-test statistics.
(DOC)

S5 Table Anticonvulsant and acute toxic effects of gabapentin (GBP), lacosamide (LCM), levetiracetam (LEV), pregabalin (PGB) and retigabine (RTG) administered singly in mice. Results are median effective doses (ED_50_ in mg/kg) and median toxic doses (TD_50_ in mg/kg) of the studied antiepileptic drugs from the 6-Hz corneal stimulation-induced seizure model and chimney or rotarod tests in mice, respectively. TI–therapeutic index is a ratio of TD_50_ and ED_50_ values. ^a^–results from this study, ^b^–results from [118], ^c^–results from [119], ^d^–results from [120], ^e^–results from [40].
(DOC)

## Abbreviations

GBP: gabapentin
LCM: lacosamide
LEV: levetiracetam
PGB: pregabalin
RTG: retigabine
