# Supplementary material for: Polygonogram and isobolographic analysis of interactions between various novel antiepileptic drugs in the 6-Hz corneal stimulation-induced seizure model in mice
Source: PLoS One. 2020 Jun 1;15(6):e0234070. doi: 10.1371/journal.pone.0234070 (PMC7263629; doi:10.1371/journal.pone.0234070)
Supplement: S1 Table — Doses of particular antiepileptic drugs used in the 6-Hz corneal stimulation-induced seizure model are placed in the first column. Results indicate numbers of animals protected from 6-Hz corneal stimulation-induced seizures per total number of animals in each experimental group. (DOC) [file pone.0234070.s001.doc]

**S1 Table. Anticonvulsant activity of gabapentin (GBP), lacosamide (LCM), levetiracetam (LEV), pregabalin (PGB) and retigabine (RTG) when administered separately in the 6-Hz corneal stimulation-induced seizure model in mice.**

| **Dose of GBP (mg/kg)** | **Number of animals** | **Total numer of animals used** |
| --- | --- | --- |
| 50 | 2/8 | 24 |
| 75 | 4/8 |
| 100 | 6/8 |

| **Dose of LCM (mg/kg)** | **Number of animals** | **Total numer of animals used** |
| --- | --- | --- |
| 3 | 3/8 | 32 |
| 5 | 4/8 |
| 10 | 6/8 |
| 15 | 7/8 |

| **Dose of LEV (mg/kg)** | **Number of animals** | **Total numer of animals used** |
| --- | --- | --- |
| 10 | 2/8 | 24 |
| 15 | 4/8 |
| 20 | 6/8 |

| **Dose of PGB (mg/kg)** | **Number of animals** | **Total numer of animals used** |
| --- | --- | --- |
| 15 | 1/8 | 24 |
| 25 | 3/8 |
| 50 | 6/8 |

| **Dose of RTG (mg/kg)** | **Number of animals** | **Total numer of animals used** |
| --- | --- | --- |
| 20 | 2/8 | 32 |
| 30 | 4/8 |
| 35 | 5/8 |
| 40 | 6/8 |

Doses of particular AEDs used in the 6-Hz corneal stimulation-induced seizure model are placed in the first column. Results indicate numbers of animals protected from 6-Hz corneal stimulation-induced seizures per total number of animals in each experimental group.
