# Supplementary material for: Polygonogram and isobolographic analysis of interactions between various novel antiepileptic drugs in the 6-Hz corneal stimulation-induced seizure model in mice
Source: PLoS One. 2020 Jun 1;15(6):e0234070. doi: 10.1371/journal.pone.0234070 (PMC7263629; doi:10.1371/journal.pone.0234070)
Supplement: S2 Table — For each tested combination of two antiepileptic drugs results indicate median effective doses (ED50 values in mg/kg ± S.E.M.) of the studied antiepileptic drugs, when administered separately, in the 6-Hz corneal stimulation-induced seizure model in mice. Test for parallelism of dose-response relationship lines for two antiepileptic drugs in the selected combinations was performed according to Litchfield and Wilcoxon [49]. In this test, if the slope function ratio (S.R.) value is higher than the factor for slope function ratio (f ratio S.R.) value, the examined two lines are non-parallel to each other [49]. On the contrary, if the S.R. value is higher than the f ratio S.R. value, the studied two lines are collateral each other [49]. n—total number of mice used at those doses whose expected antiseizure effects ranged between 4 and 6 probits; CFP–(q and p) curve-fitting parameters; q/p—ratio of q and p values; S.R.–slope function ratio; f ratio S.R.–factor for slope function ratio. N.P.—not parallel; P.–parallel. All calculations necessary to confirm the parallelism of two antiepileptic drugs’ lines were presented in more detail elsewhere [15, 24, 75, 117]. (DOC) [file pone.0234070.s002.doc]

**S2 Table. Anticonvulsant effects of gabapentin (GBP), lacosamide (LCM), levetiracetam (LEV), pregabalin (PGB) and retigabine (RTG) administered singly in the 6-Hz corneal stimulation-induced seizure model in mice.**

=================================================================================================================================

Drug combination ED_50_ (mg/kg) *n* CFP *q*/*p* S.R. f ratio S.R. parallelism

=================================================================================================================================

GBP 72.11 ± 10.79 24 2.120 (*p*) 0.501 1.770 1.679 N.P.

LCM 4.57 ± 1.44 24 1.063 (*q*) - - - -

GBP 72.11 ± 10.79 24 2.120 (*p*) 1.000 1.000 1.407 P.

LEV 14.42 ± 2.16 24 2.120 (*q*) - - - -

GBP 72.11 ± 10.79 24 2.120 (*p*) 1.123 1.154 1.444 P.

PGB 31.66 ± 7.40 16 2.380 (*q*) - - - -

GBP 72.11 ± 10.79 24 2.120 (*p*) 1.075 1.008 1.382 P.

RTG 29.03 ± 3.82 32 2.280 (*q*) - - - -

LCM 4.57 ± 1.44 24 1.063 (*p*) 1.994 1.770 1.679 N.P.

LEV 14.42 ± 2.16 24 2.120 (*q*) - - - -

LCM 4.57 ± 1.44 24 1.063 (*p*) 2.239 1.534 1.709 P.

PGB 31.66 ± 7.40 16 2.380 (*q*) - - - -

LCM 4.57 ± 1.44 24 1.063 (*p*) 2.145 1.756 1.660 N.P.

RTG 29.03 ± 3.82 32 2.280 (*q*) - - - -

RTG 29.03 ± 3.82 32 2.280 (*p*) 1.044 1.145 1.421 P.

PGB 31.66 ± 7.40 16 2.380 (*q*) - - - -

RTG 29.03 ± 3.82 32 2.280 (*p*) 0.930 1.008 1.382 P.

LEV 14.42 ± 2.16 24 2.120 (*q*) - - - -

LEV 14.42 ± 2.16 24 2.120 (*p*) 1.123 1.154 1.444 P.

PGB 31.66 ± 7.40 16 2.380 (*q*) - - - -

=================================================================================================================================

1. For each tested combination of two AEDs results indicate median effective doses (ED_50_ values in mg/kg ± S.E.M.) of the studied AEDs, when administered separately, in the 6-Hz corneal stimulation-induced seizure model in mice. Test for parallelism of dose-response relationship lines for two AEDs in the selected combinations was performed according to Litchfield and Wilcoxon [49]. In this test, if the slope function ratio (S.R.) value is higher than the factor for slope function ratio (f ratio S.R.) value, the examined two lines are non-parallel to each other [49]. On the contrary, if the S.R. value is higher than the f ratio S.R. value, the studied two lines are collateral each other [49]. n—total number of mice used at those doses whose expected antiseizure effects ranged between 4 and 6 probits; CFP–(q and p) curve-fitting parameters; q/p—ratio of q and p values; S.R.– slope function ratio; f ratio S.R.– factor for slope function ratio. N.P.—not parallel; P. – parallel. All calculations necessary to confirm the parallelism of two AEDs’ lines were presented in detail elsewhere [15, 24, 75, 117].
