# Supplementary material for: Polygonogram and isobolographic analysis of interactions between various novel antiepileptic drugs in the 6-Hz corneal stimulation-induced seizure model in mice
Source: PLoS One. 2020 Jun 1;15(6):e0234070. doi: 10.1371/journal.pone.0234070 (PMC7263629; doi:10.1371/journal.pone.0234070)
Supplement: S3 Table — Doses of particular AEDs in combination tested in the 6-Hz corneal stimulation-induced seizure model are placed in the first column. Results indicate numbers of animals protected from 6-Hz corneal stimulation-induced seizures per total number of animals in each experimental group. (DOC) [file pone.0234070.s003.doc]

**S3 Table. Anticonvulsant activity of 10 various two-drug combinations of gabapentin (GBP), lacosamide (LCM), levetiracetam (LEV), pregabalin (PGB) and retigabine (RTG) administered at the fixed-ratio of 1:1 in the 6-Hz corneal stimulation-induced seizure model in mice.**

| **Dose of LEV + RTG (mg/kg)** | **Number of animals** | **Total numer of animals used** |
| --- | --- | --- |
| LEV (0.90) + RTG (1.81) | 3/8 | 40 |
| LEV (1.34) + RTG (2.71) | 5/8 |
| LEV (1.79) + RTG (3.61) | 7/8 |
| LEV (3.60) + RTG (7.26) | 8/8 |
| LEV (7.21) + RTG (14.52) | 8/8 |

| **Dose of LEV + LCM (mg/kg)** | **Number of animals** | **Total numer of animals used** |
| --- | --- | --- |
| LEV (0.91) + LCM (0.29) | 2/8 | 40 |
| LEV (1.37) + LCM (0.43) | 4/8 |
| LEV (1.82) + LCM (0.58) | 6/8 |
| LEV (3.60) + LCM (1.14) | 8/8 |
| LEV (7.21) + LCM (2.28) | 8/8 |

| **Dose of GBP + RTG (mg/kg)** | **Number of animals** | **Total numer of animals used** |
| --- | --- | --- |
| GBP (4.49) + RTG (1.81) | 1/8 | 32 |
| GBP (8.98) + RTG (3.61) | 4/8 |
| GBP (18.04) + RTG (7.26) | 7/8 |
| GBP (36.06) + RTG (14.52) | 8/8 |

| **Dose of PGB + LEV (mg/kg)** | **Number of animals** | **Total numer of animals used** |
| --- | --- | --- |
| PGB (3.98) + LEV (1.82) | 1/8 | 24 |
| PGB (7.90) + LEV (3.60) | 3/8 |
| PGB (15.83) + LEV (7.21) | 6/8 |

| **Dose of GBP + LEV (mg/kg)** | **Number of animals** | **Total numer of animals used** |
| --- | --- | --- |
| GBP (9.00) + LEV (1.80) | 3/8 | 24 |
| GBP (18.04) + LEV (3.61) | 5/8 |
| GBP (36.06) + LEV (7.21) | 7/8 |

| **Dose of PGB + RTG (mg/kg)** | **Number of animals** | **Total numer of animals used** |
| --- | --- | --- |
| PGB (3.96) + RTG (3.64) | 2/8 | 24 |
| PGB (7.90) + RTG (7.25) | 5/8 |
| PGB (15.83) + RTG (14.52) | 7/8 |

| **Dose of PGB + LCM (mg/kg)** | **Number of animals** | **Total numer of animals used** |
| --- | --- | --- |
| PGB (4.02) + LCM (0.58) | 2/8 | 24 |
| PGB (7.91) + LCM (1.14) | 5/8 |
| PGB (15.83) + LCM (2.28) | 6/8 |

| **Dose of GBP + LCM (mg/kg)** | **Number of animals** | **Total numer of animals used** |
| --- | --- | --- |
| GBP (9.03) + LCM (0.57) | 1/8 | 24 |
| GBP (18.06) + LCM (1.14) | 4/8 |
| GBP (36.06) + LCM (2.28) | 7/8 |

| **Dose of PGB + GBP (mg/kg)** | **Number of animals** | **Total numer of animals used** |
| --- | --- | --- |
| PGB (11.87) + GBP (27.03) | 3/8 | 24 |
| PGB (15.83) + GBP (36.06) | 5/8 |
| PGB (19.78) + GBP (45.06) | 7/8 |

| **Dose of RTG + LCM (mg/kg)** | **Number of animals** | **Total numer of animals used** |
| --- | --- | --- |
| RTG (7.26) + LCM (1.14) | 2/8 | 24 |
| RTG (10.89) + LCM (1.71) | 4/8 |
| RTG (14.52) + LCM (2.28) | 6/8 |

Doses of particular AEDs in combination tested in the 6-Hz corneal stimulation-induced seizure model are placed in the first column. Results indicate numbers of animals protected from 6-Hz corneal stimulation-induced seizures per total number of animals in each experimental group.
