# Supplementary material for: Polygonogram and isobolographic analysis of interactions between various novel antiepileptic drugs in the 6-Hz corneal stimulation-induced seizure model in mice
Source: PLoS One. 2020 Jun 1;15(6):e0234070. doi: 10.1371/journal.pone.0234070 (PMC7263629; doi:10.1371/journal.pone.0234070)
Supplement: S4 Table — Both, effect size and power for each tested combination of two antiepileptic drugs were computed by a “Compromise power analysis” in G*Power software (version 3.1.9.7 for Windows–freely available) that allowed the respective calculations based on ED50, S.D. values and numbers of animals tested, as recommended elsewhere [84, 85]. In animal studies, the power equal to and higher than 0.8 (at the significance level of 0.05) is sufficiently accepted. S.D.–standard deviation; N–total number of animals used in the 6-Hz corneal stimulation-induced seizure test to calculate ED50 exp values; n–total number of animals used to calculate the ED50 add values (i.e., n = N_antiepileptic drug 1 + N_antiepileptic drug 2−4); d.f.–degree of freedom; t–critical t-test statistics. (DOC) [file pone.0234070.s004.doc]

**S4 Table. Power analysis for the tested antiepileptic drug combinations in the 6-Hz corneal stimulation-induced seizure model in mice.**

=================================================================================================================================

Drug combination ED_50 exp_ ± S.D. N ED_50 add_ ± S.D. n Effect size t d.f. Power

=================================================================================================================================

RTG+LCM 12.12 ± 8.88 24 16.80 ± 18.94 52 0.316 0.643 74 0.739

PGB+GBP 44.36 ± 22.04 24 51.88 ± 54.56 36 0.181 0.344 58 0.634

GBP+LCM 19.20 ± 16.34 8 38.34 ± 40.54 44 0.619 0.809 50 0.789

PGB+LCM 8.17 ± 11.74 24 18.11 ± 26.51 36 0.485 0.624 58 0.820

PGB+RTG 12.41 ± 13.29 16 30.34 ± 37.21 44 0.642 1.104 58 0.863

GBP+LEV 15.06 ± 20.11 16 43.27 ± 42.93 44 0.842 1.672 58 0.886

PGB+LEV 7.20 ± 9.55 16 23.04 ± 28.67 36 0.741 1.240 50 0.890

GBP+RTG 12.62 ± 10.77 8 50.57 ± 52.66 52 0.998 1.320 58 0.904

LEV+LCM 1.73 ± 1.27 24 9.50 ± 11.92 44 0.917 1.813 66 0.963

LEV+RTG 3.24 ± 2.20 24 21.73 ± 21.55 52 1.207 2.454 74 0.992

=================================================================================================================================

1. Both, effect size and power for each tested combination of two antiepileptic drugs were computed by a “Compromise power analysis” in G*Power software (version 3.1.9.7 for Windows) that allowed the respective calculations based on ED_50_, S.D. values and numbers of animals tested, as recommended elsewhere [84, 85]. In animal studies, the power equal to and higher than 0.8 (at the significance level of 0.05) is sufficiently accepted.
2. S.D. – standard deviation; N – total number of animals used in the 6-Hz corneal stimulation-induced seizure test to calculate ED_50 exp_ values; n – total number of animals used to calculate the ED_50 add_ values (i.e., n = N_antiepileptic drug 1 + N_antiepileptic drug 2 – 4); d.f. – degree of freedom; t – critical t-test statistics.
