# Supplementary material for: Polygonogram and isobolographic analysis of interactions between various novel antiepileptic drugs in the 6-Hz corneal stimulation-induced seizure model in mice
Source: PLoS One. 2020 Jun 1;15(6):e0234070. doi: 10.1371/journal.pone.0234070 (PMC7263629; doi:10.1371/journal.pone.0234070)
Supplement: S5 Table — Results are median effective doses (ED50 in mg/kg) and median toxic doses (TD50 in mg/kg) of the studied antiepileptic drugs from the 6-Hz corneal stimulation-induced seizure model and chimney or rotarod tests in mice, respectively. TI–therapeutic index is a ratio of TD50 and ED50 values. a–results from this study, b–results from [118], c–results from [119], d–results from [120], e–results from [40]. (DOC) [file pone.0234070.s005.doc]

1. **S4 Table. Anticonvulsant and acute toxic effects of gabapentin (GBP), lacosamide (LCM), levetiracetam (LEV), pregabalin (PGB) and retigabine (RTG) administered singly in mice.**

| 1. **Antiepileptic drugs** | 1. **ED_50_ (mg/kg)** | 1. **TD_50_ (mg/kg)** | 1. **TI** |
| --- | --- | --- | --- |
| 1. **Gabapentin** | 1. 72.11 ± 10.79 ^a^ | 1. 1176 (952 – 1453) ^b^ | 1. 16.3 |
| 1. **Lacosamide** | 1. 4.57 ± 1.44 ^a^ | 1. 33.77 ± 4.98 ^c^ | 1. 7.4 |
| 1. **Levetiracetam** | 1. 14.42 ± 2.16 ^a^ | 1. 1601 (1324 – 1935) ^d^ | 1. 111 |
| 1. **Pregabalin** | 1. 31.66 ± 7.40 ^a^ | 1. 144.95 ± 45.86 ^c^ | 1. 4.6 |
| 1. **Retigabine** | 1. 29.03 ± 2.25 ^a^ | 1. 38.66 ± 6.83 ^e^ | 1. 1.3 |

1. Results are median effective doses (ED_50_ in mg/kg) and median toxic doses (TD_50_ in mg/kg) of the studied antiepileptic drugs from the 6-Hz corneal stimulation-induced seizure model and chimney or rotarod tests in mice, respectively. TI – therapeutic index is a ratio of TD_50_ and ED_50_ values.
2. ^a^ – results from this study
3. ^b^ – results from [118],
4. ^c^ – results from [119],
5. ^d^ – results from [120],
6. ^e^ – results from [40].
